# Highly efficient silencing of microRNA by heteroduplex oligonucleotides

**DOI:** 10.1093/nar/gkz492

**Published:** 2019-06-19

**Authors:** Kotaro Yoshioka, Taiki Kunieda, Yutaro Asami, Huijia Guo, Haruka Miyata, Kie Yoshida-Tanaka, Yumiko Sujino, Wenying Piao, Hiroya Kuwahara, Kazutaka Nishina, Rintaro Iwata Hara, Tetsuya Nagata, Takeshi Wada, Satoshi Obika, Takanori Yokota

**Affiliations:** 1 Department of Neurology and Neurological Science, Graduate School of Medical and Dental Sciences and Center for Brain Integration Research, Tokyo Medical and Dental University (TMDU), 1-5-45 Yushima, Bunkyo-Ku, Tokyo, 113-8519, Japan; 2 Section of Molecular Technology, Core Research for Evolutional Science and Technology (CREST), Japan Science and Technology Agency (JST), 4-1-8 Honcho, Kawaguchi-shi, Saitama 332-0012, Japan; 3 Faculty of Pharmaceutical Sciences, Tokyo University of Science, 2641 Yamazaki, Noda, Chiba, 278-8510, Japan; 4 Graduate School of Pharmaceutical Sciences, Osaka University, 1-6 Yamadaoka, Suita, Osaka, 565-0871, Japan

## Abstract

AntimiR is an antisense oligonucleotide that has been developed to silence microRNA (miRNA) for the treatment of intractable diseases. Enhancement of its *in vivo* efficacy and improvement of its toxicity are highly desirable but remain challenging. We here design heteroduplex oligonucleotide (HDO)-antimiR as a new technology comprising an antimiR and its complementary RNA. HDO-antimiR binds targeted miRNA *in vivo* more efficiently by 12-fold than the parent single-stranded antimiR. HDO-antimiR also produced enhanced phenotypic effects in mice with upregulated expression of miRNA-targeting messenger RNAs. In addition, we demonstrated that the enhanced potency of HDO-antimiR was not explained by its bio-stability or delivery to the targeted cell, but reflected an improved intracellular potency. Our findings provide new insights into biology of miRNA silencing by double-stranded oligonucleotides and support the *in vivo* potential of this technology based on a new class of for the treatment of miRNA-related diseases.

## INTRODUCTION

MicroRNAs (miRNAs) are endogenous small non-coding RNAs (21–23 nucleotides) that inhibit mRNA post-transcriptionally (1) and play crucial roles in various physiological processes and responses to pathogens (2). Increasing evidence indicates that various human diseases are caused by altered expression and profiling of miRNAs (3,4). Hence, targeting of specific miRNAs will produce novel therapeutic strategies (4–6). One promising approach to inhibit miRNA is antisense oligonucleotides (ASO), known as antimiR (7–9).

Steric-blocking and degradation of target RNA are major silencing mechanisms of ASO including antimiR (7,10,11). Steric-blocking type of ASO binds and sequesters its target RNA in duplexes, while degradation-type of ASO induces degradation of its target RNA. For therapeutic standpoint, RNA-degradation mechanism has potential advantages over steric-blocking. One of the advantages is higher turnover ratios of silencing (12,13). While steric-blocking type of antimiRs bind and sequester single target miRNAs in RNA induced silencing complexes (RISC) and are not subsequently recycled, degradation-type of antimiRs can bind and be released from target miRNAs multiple times, allowing greater turnover ratios (7,8,14). In addition, degradation-type of antimiRs have lower cell toxicity, because degradation of the targeted miRNA allows RISC to be recycled and remain functional. In contrast, duplexes of steric-blocking type of antimiR and target miRNAs may occupy RISC, causing cell toxicity by interfering with the maturation of other miRNAs (10).

Relationship between antimiR-chemistry and miRNA-inhibition mechanisms has been demonstrated in recent studies (7,10,15). To improve bio-stability and binding affinity for target miRNA, antimiR requires chemical modifications including phosphorothioate (PS) bonds in internucleotide linkages (16) and sugar modifications, such as 2′-*O*-methyl RNA (2′OMe) (9,17) or locked nucleic acid (LNA) (18,19). AntimiRs with lower-affinity chemical modifications, such as 2′OMe, induce miRNA-degradation (20–22), while antimiRs with higher-affinity chemical modifications, such as LNA, do not induce miRNA degradation but inhibit its target miRNA by steric-blocking mechanism (15,23). Since high affinity of LNA chemistry enables efficient miRNA-silencing *in vivo* (18,24–26), LNA-modified antimiRs have been tested in clinical trials (27–29). However, improvements of *in vivo* potency and toxicities are highly desirable to accommodate high synthetic costs and to avoid adverse effects. On the basis of above advantages of degradation-mechanism, development of degradation type LNA-antimiR is promising approach (30).

We recently developed DNA–RNA heteroduplex oligonucleotides (HDO) which comprise a DNA/LNA gapmer type of ASO that target messenger RNA (mRNA) and its complementary RNA (cRNA) strand as a novel class of ASO (31). After a lipid ligand such as alpha-tocopherol (Toc) conjugation with the cRNA strand, HDO produced improvements in mRNA silencing effects in the liver.

In this study, we designed double-stranded HDO-antimiRs and demonstrated improvements of *in vivo* potency compared with the parent antimiR. The present data reveal that HDO-antimiRs with LNA-chemistry can enhance the potency of intracellular miRNA-silencing.

## MATERIALS AND METHODS

### Design and synthesis of antisense oligonucleotides

AntimiRs were designed to target miR-122 or -21 based on a previous report (18) and were synthesized by GeneDesign Inc. (Osaka, Japan). Detailed sequence information is shown in Supplementary Table S1. A series of cRNAs was synthesized by Hokkaido System Science (Sapporo, Japan). Cy5 or Cy3 fluorophores were covalently bound to the 5′ or 3′ ends of DNA/LNA antimiRs or cRNAs. Alpha-tocopherol was covalently conjugated with 5′ ends of DNA/LNA antimiRs or cRNAs and three phosphodiester-linked monovalent *N*-acetylgalactosamine (GalNAc) units were tethered to 5′ ends of antimiRs or cRNAs as reported in a previous report (32). To generate HDO, equimolar concentrations of antimiR and cRNA strands were heated in PBS (Sigma-Aldrich) at 95°C for 5 min and were then cooled to room temperature over 1 h.

### Mouse studies

Wild-type female Crlj: CD1 (ICR) mice at 4–5 weeks of age were obtained from Oriental Yeast Co., Ltd (Tokyo, Japan). AntimiRs were administered to mice (*n* = 4 or 5 per group) according to body weights using tail vein injections. All oligonucleotides were formulated in PBS, which was also used as the control. Prior to postmortem analyses, mice were anesthetized with intraperitoneal injections of 60 mg/kg pentobarbital and were then euthanized by transcardiac perfusions with PBS. All protocols met ethics and safety guidelines for animal experimentation and were approved by the ethics committee of Tokyo Medical and Dental University (#0170179A).

### Quantitative RT-PCR

Total RNA was isolated using a MagNA Pure 96 system (MagNA Pure 96 Cellular RNA Large Volume Kit, Roche Diagnostics). cDNAs were synthesized using TaqMan miRNA assays (Applied Biosystems) for miRNA experiments and detection for antimiR, or using Transcriptor Universal cDNA Master (Roche) for mRNA experiments. Subsequent qRT-PCR analyses were normalized to U6 small RNA for miRNA experiments or to *Actb* (beta-actin) mRNA for mRNA experiments and were performed using TaqMan primers (Applied Biosystems, Supplementary Table S2) in a LightCycler 480 System with a LightCycler 480 Probes Master kit (Roche). All the studies were performed in accordance with Minimum Information for publication of quantitative real-time PCR Experiments (MIQE) guidelines (33).

### Determination of antimiR concentrations in biological samples

Serum and tissue samples were obtained from mice after intravenous injections with Cy5–labeled antimiRs (serum and liver, 24 or 180 nmol/kg; kidney and spleen, 180 nmol/kg) at each time point. Tissues were homogenized in 250 μl of PBS (Sigma-Aldrich) and Cy5 concentrations were measured using Infinite M1000 Pro (Tecan, Männedorf, Switzerland).

### Analysis of distributions and localizations of antimiRs in liver tissues

Mice were injected with 180 nmol/kg Cy5–labeled antimiR or HDO-antimiR, were fixed using 4% paraformaldehyde in PBS for 12 h, and were then snap-frozen in liquid nitrogen. Liver tissues were then collected and tissue sections (10 μm) were prepared using a cryostat (model CM3050 S, Leica Microsystems). Sections were stained with DAPI (Vector Laboratories) to visualize nuclei and with 13 nmol/l AlexaFluor 488 phalloidin (Life Technologies) to visualize cell membranes. Stained sections were then analyzed using laser scanning confocal microscopy (model A1R, Nikon, Japan).

### Isolation of hepatocytes and non-parenchymal cells

Hepatocytes and non-parenchymal cells were isolated from mice livers following a collagenase digestion as previously described (34). Briefly, liver tissues were perfused with EGTA buffer and subsequently dissociated with 0.05% collagenase at 37°C. Hepatocytes were separated from the whole liver fractions by five low-speed centrifugations (50 g for 1 min). Non-parenchymal cells were collected by centrifugation (15 000 rpm for 7 min).

### Cell transfection

For transfection with antimiRs by lipid, Huh-7 cells were transfected in 24-well plates with increasing concentrations of antimiR in Opti-MEM containing 10 μl/ml Lipofectamine RNAiMAX (Life Technology) without serum. After 4 h, transfection media were replaced with complete medium comprising DMEM (Invitrogen) and were incubated for another 20 h before use in subsequent experiments. For transfection with naked antimiRs, Hepa 1-6 cells were transfected in 48-well with increasing concentrations of antimiR with or without 30% mouse serum from ICR mice. After 48 h, the cells were harvested and used in subsequent experiments.

### Electrophoretic mobility shift assay (EMSA)

Cy3–labeled parent antimiR or HDO-antimiRs (5 pmol) were added to 1.0–7.5 μl aliquots of mouse serum, and quarter volumes of 10% sucrose were then added. Samples were resolved using electrophoresis in 2% agarose gels in Tris-borate-EDTA buffer for 20 min at 100 V. Finally, oligonucleotides were visualized under UV light.

### Northern blotting analysis

Northern blotting analyses of miRNAs or the overhang of a 35-mer HDO-antimiR cRNA-strand were performed as previously reported (35) with slight modifications. Briefly, total RNA was extracted from mouse livers using Isogen II (Nippon Gene) and 4 μg of miR-122, 25 μg of miR-21 or cRNA, or 0.1 pmol size markers were separated using electrophoresis in 15% or 20% (high-resolution northern blots) polyacrylamide–24% urea gels and were then transferred to Hybond-N+ membrane (Amersham Biosciences, Piscataway, NJ). The miRCURY LNA Detection Probe was used to detect miRNA (Exiqon). Probes for U6 or cRNA (5′-TGGTGCGTATGCGTAGCATTGGTATTCA-3′) were labeled with digoxigenin-ddUTP (DIG Oligonucleotide 3′-End Labeling Kit, 2nd Generation, Roche Diagnostics). Signals were visualized using Gene Images CDP-star Detection Kits (Amersham Biosciences), and band intensities were analyzed using Image Lab software version 5.2.

### Confocal imaging of Cy5/Cy3 labeled HDO-antimiR

Hepa 1–6 cells were seeded in collagen type I cell ware 4-well culture slide (BD BioCoat) and maintained for 24 h. HDO-antimiR labeled with fluorescence was prepared with annealing as follows; with antimiR strand labeled by Cy5 and cRNA strand labeled by Cy3 each. This HDO-antimiR was transfected to the cells by gymnotic delivery with media 10% mouse serum at a final concentration of 500 μM. After 6 h incubation, cells were washed and stained with DAPI (Sigma-Aldrich) to visualize nuclei and with AlexaFluor 488 phalloidin (Life Technologies) to visualize cell membranes. Confocal images were acquired by model A1R laser scanning confocal microscopy (NIKON). A fluorescence signal of Cy5-antimiR strand was collected by excitation at 647 nm and emission collection by using band pass 663–738 nm, and a fluorescence signal of Cy3-antimiR strand was collected by excitation at 560 nm and emission collection by using band pass 570–620 nm. Fluorescence resonance energy transfer (FRET) signal was collected by excitation at 560 nm and emission collection by using band pass 663–738 nm.

### Separation of RNA samples to release miRNA from antimiR

RNA samples of 30-μg were incubated with RNase-Free DNase I (Sigma-Aldrich) at 37°C for 1 h according to respective protocols, were denatured at 95°C and were then immediately cooled on ice.

### Statistical analysis

Animal experiments were performed with four or five mice for each treatment group. Pairwise comparisons were performed using Student's *t* test and multiple comparisons were performed using one-way ANOVA with Bonferroni's multiple-comparison test. Differences were considered significant when *P* < 0.05 and all statistical analyses were performed using Prism version 6.05 (GraphPad Software).

## RESULTS

### Enhanced miRNA-silencing potency of HDO-antimiR *in vivo*

**Figure 1.**
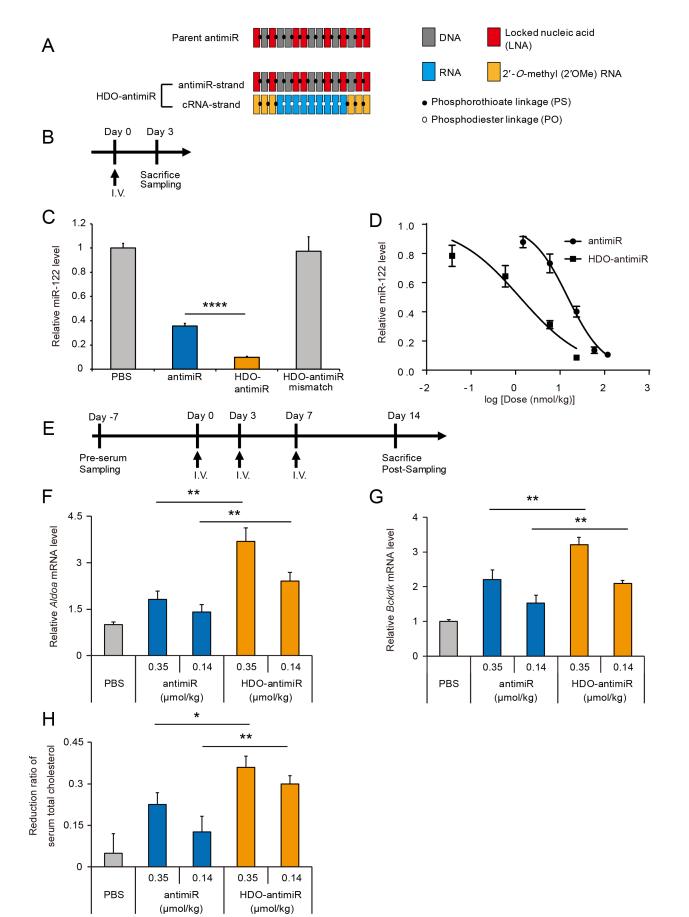
Enhanced in vivo potency of miRNA-silencing by heteroduplex oligonucleotide (HDO)-antimiR. (**A**) Design of a DNA/LNA mixmer-type of antimiR and HDO-antimiR. (**B**) Experimental design for single-injection study. (**C**) Quantitative RT-PCR (qRT-PCR) analysis of relative miR-122 expression in livers from mice treated with the parent antimiR, HDO-antimiR, an HDO-antimiR mismatch, or phosphate buffered saline (PBS) at 24 nmol/kg (corresponding to 0.1 mg/kg as the parent antimiR). (**D**) Dose–response curve of miR-122 inhibition in livers of mice treated with antimiRs. (**E**) Experimental design for three-injection study. (**F, G**) qRT-PCR analyses of relative *aldolase A (Aldoa*) and *branched chain keto acid dehydrogenase kinase* (*Bckdk*) mRNA expression levels which were suppressed by miR-122 in mouse livers after treatment with antimiRs at 0.35 or 0.14 μmol/kg (corresponding to 1.5 or 0.6 mg/kg as the parent antimiR). (H) Reduction ratios of serum total cholesterol relative to those before injections in the same animals (F, G). Mean values ± SEM (*n* = 5 except for 5.9 and 24 nmol/kg groups in B; *n* = 9); *P < 0.05, **P < 0.01 and ****P < 0.0001; multiple comparisons were performed using one-way ANOVA with Bonferroni's test.

To investigate whether the HDO-structure can enhance silencing of miRNA by antimiR, we designed an HDO-antimiR comprising an antimiR-strand targeting miRNA-122 (miR-122) and its cRNA-strand (Figure 1A). The antimiR-strand is a 15-mer DNA/LNA mixmer-type antisense oligonucleotide with a complete PS backbone and totally same as the most advanced antimiR (miravirsen) with steric-blocking mechanism previously reported (18). Both 3′- and 5′-wing portions of the cRNA-strand comprise three 2′OMe nucleotides with PS modifications.

Initially, we examined *in vivo* miRNA silencing following single intravenous administration of HDO-antimiR to mice and made comparisons with the parent antimiR (Figure 1B). HDO-antimiR bound endogenous miR-122 in the liver more efficiently than the parent single-strand antimiR (Figure 1C). In these experiments, we used an HDO-antimiR with two LNA-nucleotide mismatches of the miR-122 sequence as a negative control (18), and observed no binding of miRNA. These data demonstrate that HDO-antimiR binds targeted miRNA in a sequence-dependent manner. In addition, dose-response curve analyses (Figure 1D) revealed dose dependent miRNA-binding by the parent antimiR and HDO-antimiR, and showed 12-fold greater binding efficiency by HDO-antimiR compared to the parent antimiR (50% binding-dose by HDO-antimiR or the parent antimiR: 1.3 versus 15 nmol/kg respectively).

In further studies, we examined the effects of conjugation with the liver delivery molecule Toc. In our previous report, direct Toc-conjugation to mRNA targeted ASO interfered with mRNA silencing effects (31). In contrast, direct Toc-conjugation to antimiR enhanced miRNA silencing (Supplementary Figure S1). Surprisingly, suppression of miRNA by HDO-antimiR was superior to that by the Toc conjugated single-stranded antimiR, and was further amplified by Toc-conjugation to the cRNA-strand.

To further assess potency of HDO-antimiR, we evaluated its efficacy in increasing expression of mRNAs which were directly suppressed by target miRNAs. After administering three repeated injections in a week (Figure 1E), the HDO-antimiR against miR-122 dose-dependently derepressed the miR-122 targeted mRNAs *Aldoa* and *Bckdk* (9,18,25) in liver tissues more efficiently than the parent antimiR (Figure 1F and G). Similarly, treatments with an HDO-antimiR against miR-21 led to improved upregulation of the corresponding mRNAs *Taf7* and *Spg20* (36) in the liver (Supplementary Figure S2). Moreover, at higher doses, this HDO-antimiR showed enhancement of the mRNA upregulation in extrahepatic tissues such as the kidney, spleen, and adrenal gland (Supplementary Figure S3).

To test the phenotypic effects of antimiRs against miR-122, we evaluated serum total cholesterol levels, which were decreased due to inhibition of miR-122 in liver. As shown in Figure 1H, HDO-antimiR reduced serum total cholesterol levels more effectively than the parent antimiR.

To assess hepatic or renal toxicity of HDO-antimiR, we performed serum biochemical analyses of mice at seven days after three repeated intravenous injections in one week (Table 1 and Supplementary Table S3). These analyses demonstrated that HDO-antimiR did not induce hepatotoxicity or renal toxicity, whereas the parent antimiR increased serum creatinine and urea nitrogen levels at the same dose.

**Table 1.** Serum, liver and kidney parameters of mice treated with PBS (control), antimiR or HDO-antimiR

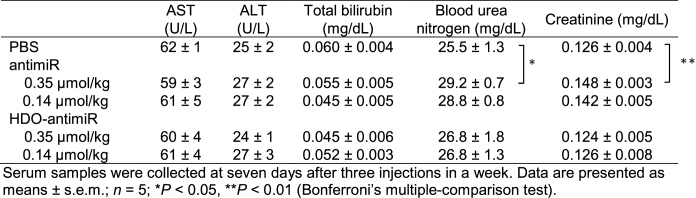

### Biodistribution of HDO-antimiR

**Figure 2.**
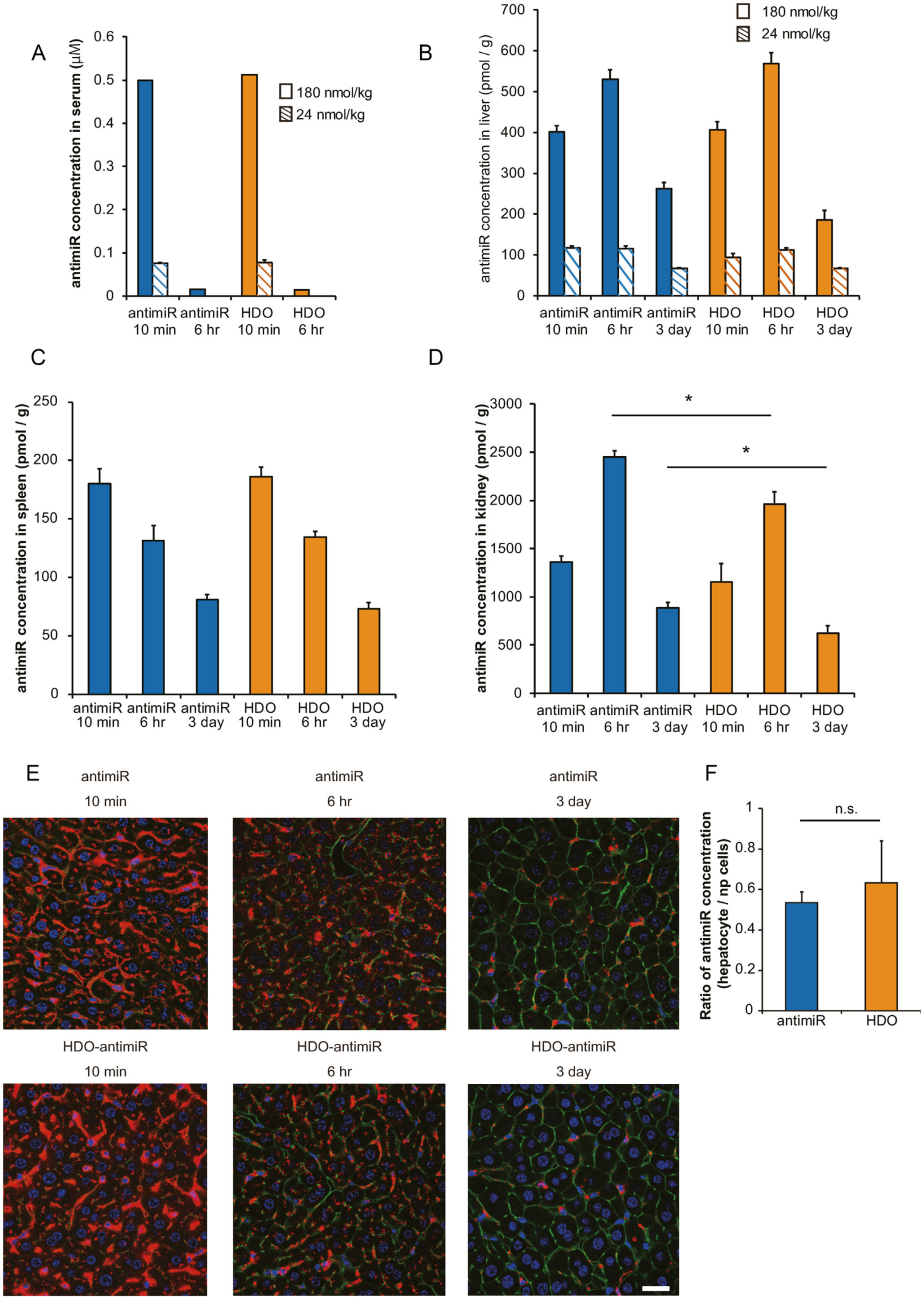
Pharmacokinetics and biodistributions of HDO-antimiR. (A–D) AntimiR concentrations in serum (**A**), liver (**B**), spleen (**C**) and kidney (**D**) tissues of mice (*n* = 4) treated with single intravenous injections of Cy5-labeled antimiRs at 24 or 180 nmol/kg; no fluorescence was detected in the serum at 6 h after 24 nmol/kg injections. (**E**) Confocal laser scanning microscopic images of livers from mice treated with Cy5-labeled antimiRs at 180 nmol/kg; Cy5-labeled antimiR [red]; AlexaFluor 488 phalloidin [green]; DAPI [blue]; bar = 25 μm. (**F**) Differential accumulation of antimiRs (*n* = 3) between hepatocytes and non-parenchymal (np) cells. Mice were sacrificed at 3 days after single 24 nmol/kg intravenous injections of antimiRs. Liver tissues were fractionated into hepatocytes and np cells and assayed for antimiR accumulation ratio of hepatocytes to np cells by qRT-PCR. Mean values ± SEM; *P < 0.05, **P < 0.01; multiple comparisons were performed using one-way ANOVA with Bonferroni's test.

To investigate the mechanisms behind enhanced *in vivo* miRNA silencing by HDO-antimiR, we evaluated the concentrations of HDO-antimiR in serum and tissues compared to those of the parent antimiR. Cy5–labeling of the antimiR-strand revealed similar concentrations of the parent antimiR and HDO-antimiR including single-stranded and duplex in serum, liver, and spleen (Figure 2A–C) after single intravenous injections. In contrast, HDO-antimiR concentrations in the kidney were less than those of the parent antimiR (Figure 2D). Moreover, after treatments with Cy5–labeled antimiR or HDO-antimiR, confocal laser scanning microscopy analyses of liver sections revealed similar histological distributions (Figure 2E). To investigate whether HDO-antimiR makes a shift in accumulation from non-parenchyma (np) cells into hepatocytes compared with the parent antimiR, we evaluated ratios of antimiR concentration in hepatocytes compared to that in non-parenchymal cells from mice treated by antimiRs. As a result, there was no significant difference between the ratios in the parent antimiR group and the HDO-antimiR group (Figure 2F). In addition, we evaluated *in vivo* potency of antimiRs conjugated with GalNAc, which is well-defined hepatocyte-targeted ligand based on binding to hepatocyte-specific asialoglycoprotein receptor (37–40). This analysis showed that HDO-antimiR conjugated with GalNAc was more potent in liver than the parent antimiR conjugated with GalNAc (supplementary Figure S4), demonstrating that HDO-antimiR maintained the superiority of intracellular potency compared to the single-stranded antimiR in spite of hepatocyte specific distribution by GalNAc-conjugation. Thus, neither bio-stability characteristics of HDO-antimiR in serum and tissues nor biodistribution were related to its high miRNA-silencing potency in liver, kidney and spleen tissues.

### Effects of chemical modifications in the cRNA-strand of HDO-antimiR on miRNA silencing and binding to serum molecules

**Figure 3.**
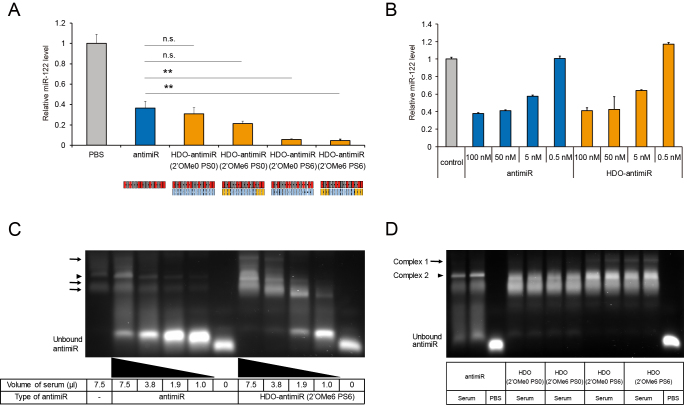
Effects of chemical modifications in the cRNA-strand on miRNA silencing and patterns of serum molecule binding. (**A**) qRT-PCR analysis of miR-122 in liver tissues from mice treated with 24 nmol/kg injections of the parent antimiR or HDO-antimiRs with varying chemical modifications at both ends of the cRNA-strand; mean values ± SEM (*n* = 5); *P < 0.05; multiple comparisons were performed using one-way ANOVA with Bonferroni's test. (**B**) qRT-PCR analysis of miR-122 expression in Huh-7 cells at 24 h after treatment with increasing concentrations of antimiR or HDO-antimiR without serum (*n* = 4). (**C**) EMSA after incubation with increasing concentrations of mouse serum show serum protein binding by Cy3–labeled parent antimiR or HDO-antimiR (2′OMe6 PS6) with six 2′OMe and six PS modifications at both ends of the cRNA-strand; positions of bands corresponding to complexes of serum molecules with parent antimiR or HDO-antimiR are indicated by arrow heads and arrows, respectively. (**D**) EMSA analysis of binding patterns between the Cy3-labeled antimiRs and 7.5 μl serum are presented as in Figure 3A.

To investigate contributions of chemical modifications in the cRNA-strand of HDO-antimiR to silencing effects, we assessed miRNA suppression by HDO-antimiRs with differing numbers of PS and 2′OMe modifications at the wing portions of the cRNA-strands. This study revealed that PS modifications improved the suppressive effects of miRNA by HDO-antimiR more effectively than 2′OMe modifications (Figure 3A).

Multiple previous studies show that PS modifications improve resistance to nucleases (16,41) and protein binding affinity (42–44). Recent studies also reveal that profiling and affinity of ASO-carrier proteins in serum can affect cellular uptake and subsequent subcellular trafficking, hence influencing intracellular potency of ASOs (45,46).

To determine the effects of binding to serum molecules on HDO-antimiR potency, we performed *in vitro* assays with transfection of antimiRs into cells by liposome mediated reagent without serum protein binding. These assays showed similar potency of HDO-antimiR and the parent antimiR (Figure 3B). Moreover, we assessed *in vitro* potency of the single-stranded or HDO-antimiR without any transfection reagents under the presence or absence of serum. This assessment showed that only HDO-antimiR with serum was able to increase the *Spg20* and *Taf7* mRNA which were suppressed by the targeted miR-21 (Supplementary Figure S5). These findings suggested that HDO-antimiR has increased potency in cells after binding to serum carrier molecules.

To examine binding patterns between serum carrier molecules and HDO-antimiR, we performed electrophoretic mobility shift assays (EMSA). In these assays, the parent antimiR formed a single complex (arrow head in Figure 3C), whereas the HDO-antimiR formed three additional complexes (arrows in Figure 3C) during incubation in mouse serum. Moreover, compared with the parent antimiR, the lowest bands of unbound and free HDO-antimiR were weaker, in contrast the higher bands of HDO-antimiR bound to serum molecules were stronger (Figure 3C). These differing binding patterns and higher affinities of HDO-antimiR were also observed in serum from intravenously treated mice (Supplementary Figure S6) and higher affinities of HDO-antimiR was confirmed in human serum analysis (Supplementary Figure S7). These findings indicated that HDO-antimiR binds serum molecules more effectively than the parent antimiR *in vivo* and formed additional complexes in the serum.

Finally, we investigated the effects of differing numbers of chemical modification in the cRNA-strand of HDO-antimiRs (Figure 3A) on binding serum molecules. Regardless of chemical modifications in cRNA-strands, all HDO-antimiRs similarly showed higher binding affinity for serum molecules (Figure 3D). However, formation of complex 1 was dependent on PS modifications, but not on 2′OMe modifications at the wings of the cRNA-strand (Figure 3D). These EMSA analyses indicated that enhancements of *in vivo* miRNA-silencing potency may be associated with the unique complexes between serum molecules and efficient HDO-antimiRs with PS modifications in the cRNA-strand.

### Intracellular trafficking and cleavage of the cRNA-strand of HDO-antimiR

**Figure 4.**
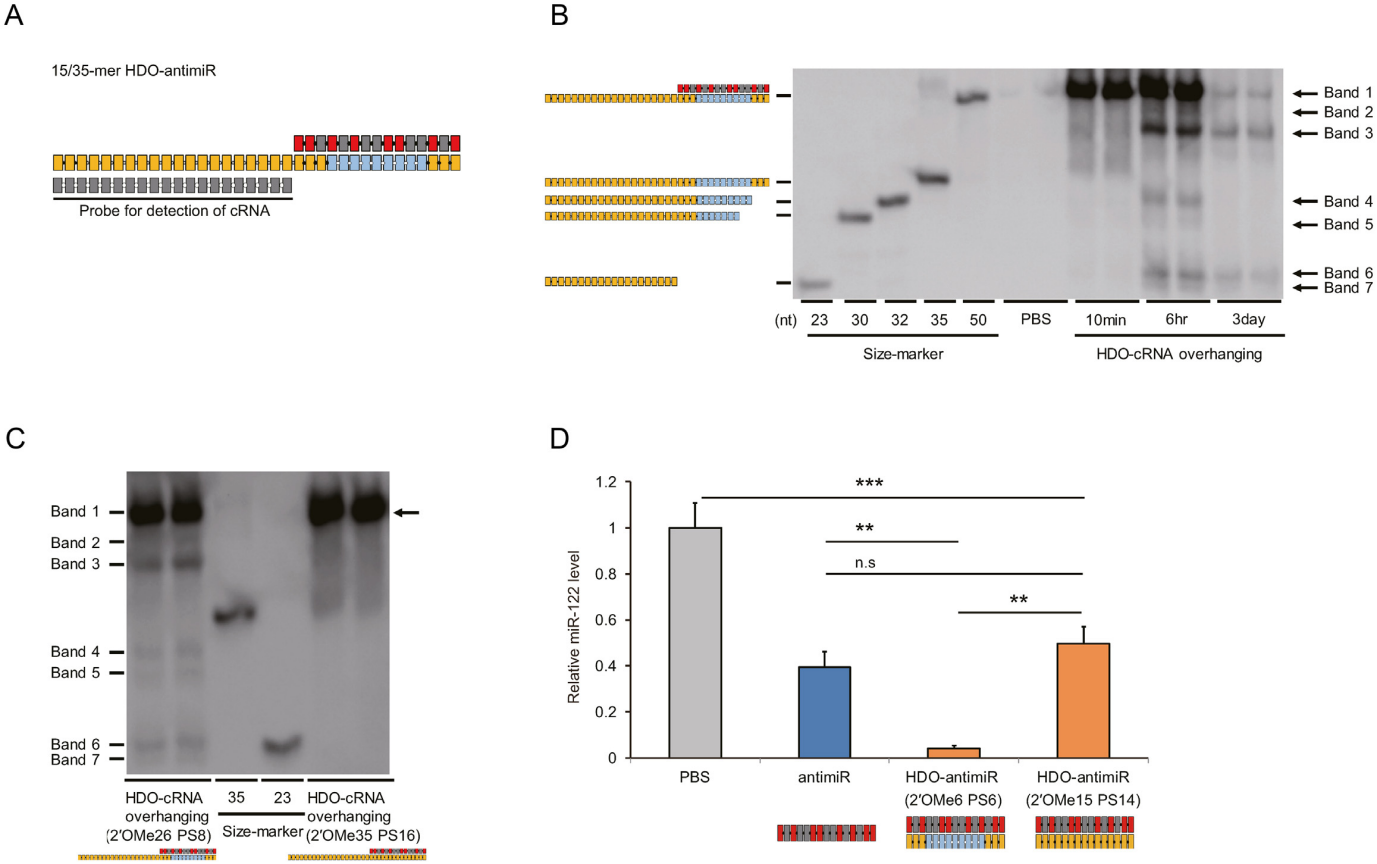
Intracellular cleavage and unwinding of the cRNA-strand. (**A**) Design of the DNA/LNA antimiR/35-mer cRNA with fully 2′OMe- and PS-modified overhangs. (**B**) Northern blotting analyses using a probe for the overhanging cRNA portion of the 35-mer cRNA HDO-antimiR in mouse livers at 10 min, 6 h or 3 days after single injections of PBS or the 35-mer cRNA HDO-antimiR at 350 nmol/kg (*n* = 2); structures of size markers are illustrated in left panels. (**C**) Northern blotting analyses of cRNA in RNA samples at six h after injections of the 35-mer cRNA HDO-antimiR (2′OMe26 PS8) shown in Figure 4B or a 35-mer cRNA HDO-antimiR (2′OMe35 PS16) in which the cRNA-strand was fully modified with PS and 2′OMe at 350 nmol/kg; in the right blot, the complex of 35-mer cRNA and the antimiR-strand (arrow) are indicated. (D) qRT-PCR analyses of relative miR-122 levels in livers from mice treated with the parent antimiR, HDO-antimiR (2′OMe6 PS6), or HDO-antimiRs in which the cRNA-strand was fully modified with PS and 2′OMe at 24 nmol/kg; mean values ± SEM (*n* = 5); **P < 0.01, ***P < 0.001; n.s., not significant; multiple comparisons were performed using one-way ANOVA with Bonferroni's test.

To confirm that the cRNA strand of HDO-antimiR is impervious to intracellular cleavage, we performed northern blotting analysis of liver RNA samples from mice after treatments with a modified HDO-antimiR carrying a longer 35-mer cRNA-strand, which was detectable by a probe (Figure 4A). Initially, we confirmed that potency of this 15/35-mer HDO-antimiR were similar to those of the original 15/15-mer HDO-antimiR *in vivo* (Supplementary Figure S8).

cRNA-strand was cleaved into several fragments (bands 4–7 in Figure 4B) at 6 h after injection and most cRNA-strand was degraded over three days. In this figure, double-stranded HDO-antimiR is represented by bands 1–3 and unwound cRNA is represented in bands 4–7, indicating that the cRNA-strand was cleaved without unwinding. To determine how unwinding of the cRNA-strand affected the potency of miRNA silencing, we fully replaced the unmodified nucleotides of the center portion in the cRNA-strand with an RNase-resistant 2′OMe. Northern blot detecting cRNA showed that duplex of HDO-antimiR with the 2′OMe-modified cRNA remained more abundant than that of unmodified HDO-antimiR (arrow in Figure 4C), indicating that unwinding of the 2′OMe-modified cRNA is slower than the unmodified cRNA. In subsequent experiments, silencing of miR-122 by the HDO-antimiR with the 2′OMe-modified cRNA-strand was reduced markedly compared with that of the original HDO-antimiR (Figure 4D). However, both HDO-antimiRs showed the same serum binding profile in EMSA experiments (Supplementary Figure S9). These results indicate that slow unwinding of the cRNA may affect the present enhancements of miRNA silencing independently of serum binding. Moreover, HDO-antimiR with 2′OMe-modified cRNA inhibited miR-122 with the same potency as the parent antimiR, indicating that unwinding of the 2′OMe-modified cRNA can occur.

Because fluorescence resonance energy transfer (FRET) technology has been successfully applied to discriminate between single- or double-stranded siRNAs (47–49), we performed FRET-based imaging analysis to reveal intracellular localization of intact duplex HDO-antimiR. We tested HDO-antimiRs which are tagged with Cy5 to antimiR-strand and Cy3 to cRNA-strand respectively (Supplementary Figure S10). In HDO-antimiR tagged with both antimiR- and cRNA strands, Cy5 and Cy3 are < 10nm apart similarly to siRNA and a FRET signal of Cy5 should be detected at 670 nm upon excitation of the donor Cy3 which leads to energy transfer and emission from the acceptor Cy5 (47–50). Actually, we found that a reliable FRET signal of HDO-antimiR was detected (Supplementary Figure S10A). FRET signals representing duplex HDO-antimiR were present in not only cytoplasm (arrowhead in Supplementary Figure S10A) but also in nucleus (arrow in Supplementary Figure S10A), especially appeared to be localized to nucleolus, which is excluded by DAPI staining (arrows in Supplementary Figure S10B) (51,52). In addition, Cy5 signals from HDO-antimiR by 647 nm excitation which represented both the duplex and single-stranded antimiR strand separated from the duplex (Supplementary Figure S10C) was observed in cytoplasm more abundantly than FRET signal, suggesting that unwinding of HDO-antimiR occurs in cytoplasm. These findings indicated that some HDO-antimiR unwind in cytoplasm and other intact duplex HDO transport from cytoplasm into nucleus without unwinding.

### Intracellular mechanism of miRNA silencing by HDO-antimiR

**Figure 5.**
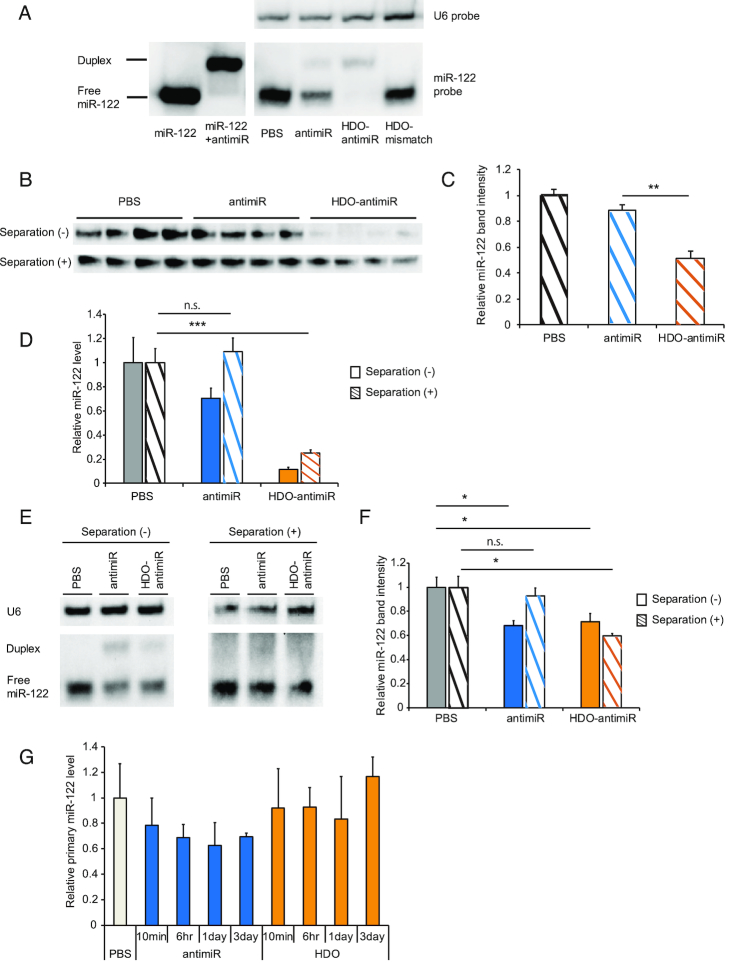
Intracellular miRNA-silencing mechanism of HDO-antimiR. (**A**) Size markers and RNA samples (n = 1) from the livers of the mice in Figure 1B were analyzed using northern blotting with a miR-122–specific probe. (**B**) Northern blotting of miR-122-inhibition with or without the separation-procedure to release miR-122 from antimiRs in RNA samples (*n* = 4) from the mice at 24 nmol/kg in Figure 1B. (**C**) Analysis of free miR-122 band intensities after the procedure in (B). (**D**) qRT-PCR analysis (*n* = 5) of miR-122-inhibition with or without the separation-procedure to release miR-122 from antimiRs from RNA samples in Figure 1B. (**E**) Northern blotting with or without the separation-procedure in RNA samples (*n* = 1) from mice at three days after single injection of the parent- or HDO-antimiR at ED_50_ dose (15 versus 1.3 nmol/kg, respectively). (**F**) Analysis of free miR-122 band intensities using same RNA samples as (E) (*n* = 4). (**G**) qRT-PCR analysis of primary-miR-122 in livers of mice at 10 min, 6 h, 1 day or 3 days after single injections of the parent antimiR or HDO-antimiR at 24 nmol/kg. Mean values ± SEM; n.s.: not significant, *P < 0.05, ***P < 0.001; multiple comparisons were performed using one-way ANOVA with Bonferroni's test.

To further investigate intracellular mechanism of enhanced miRNA inhibition by HDO-antimiR, we performed northern blotting analysis of miR-122 in liver tissues from mice treated with HDO-antimiR (Figure 1C). Both parent antimiR and the antimiR-strand of HDO-antimiR formed duplexes with miR-122 (Figure 5A), as demonstrated by decreased free miR-122 after treatment with the parent antimiR, and almost no free miR-122 after treatment with HDO-antimiR. Surprisingly, total intensity of the two bands representing free and duplex miR-122 decreased after treatment with HDO-antimiR more efficiently than that after treatment with the parent antimiR. Similar result was also observed by the HDO-antimiR targeting miR-21 (Supplementary Figure S11). To confirm whether HDO-antimiR reduced total miR-122 contents, we assessed expression level of miR-122 after separating antimiR from miRNA by DNase-degradation and heating. Also, after the separation, HDO-antimiR decreased total miR-122 contents in both northern blots (Figure 5B and C) and qRT-PCR analyses (Figure 5D). In addition, we assessed total miR-122 contents in livers from mice treated by the parent antimiR and HDO-antimiR at the 50% binding dose by qRT-PCR (Figure 1D) respectively, so that both miR-122 detection levels should be same. In Figure 5E and F, both antimiRs decrease the detection of miR-122 without separation of antimiR from miRNA by almost same level. In contrast, not the single-stranded antimiR but only HDO-antimiR was able to decrease the detection of total miR-122 with the separation. These results demonstrated that the degradation of the target miR by HDO-antimiR depends on not the high potency but its double-stranded structure. Because a recent study showed that DNA/LNA mixmer type of antimiR suppressed primary transcripts of mature-miRNA by *in vitro* assessments (53), we evaluated *in vivo* expression of primary-miR122 after treatment with HDO-antimiR. However, neither the parent antimiR nor the HDO-antimiR inhibited primary miR-122 in liver (Figure 5G). These results indicated that HDO-antimiR could efficiently access and bind mature miRNA and decrease total concentrations of target mature-miRNA molecules without influencing primary miRNA.

## DISCUSSION

HDO-antimiR has a unique double-stranded structure comprising a DNA/LNA mixmer type of antimiR and its complementary RNA, which is totally different from previously described antimiRs (54,55). We achieved a 12-fold increase in potency of *in vivo* miRNA binding by HDO-antimiR compared with the parent antimiR (Figure 1). This finding is surprising because previous studies demonstrated that HDO targeting ‘messenger RNA’ without a ligand-conjugation to complementary strand cannot improve the potency of mRNA silencing (31,40). Moreover, bio-stability and delivery ability to the targeted cells were not improved compared with those of the parent antimiR (Figure 2), indicating that the greater miRNA-silencing potency of HDO-antimiR reflects an improved intracellular potency after uptake into cell.

The current study demonstrated that major intracellular processing step of HDO-antimiR *in vivo* involves unwinding with the cleavages of the cRNA-strand (Figure 4B). FRET-based imaging showed that intact duplex HDO-antimiRs are present not only in cytoplasm but also within nucleus, indicating that the some duplex HDO-antimiR may escape from endo-lysosomal compartments, transporting into nucleus without unwinding or unwound in the cytoplasm. Especially, there is a possibility that Argonaute 2 (Ago2) may recognize and cleave HDO-antimiR since previous studies reported that Ago2 recognises 16-nt RNA duplex (56) and a variety of chemically modified miRNA mimics (57–59).

Most interesting finding in this study was that HDO-antimiR decreased contents of total miRNA which included free and bound to the antimiR although the parent antimiR did not decrease (Figure 5A–F). In experiments with the parent antimiR, total miRNA expression levels of free and bound miR-122 were similar to those of endogenous miRNA in negative controls, indicating that the inhibitory mechanism of antimiR was steric blocking. In contrast, total expression levels of miR-122 were markedly decreased by HDO-antimiR compared with the control, suggesting that in addition to steric blocking activities, HDO-antimiR downregulated mature miRNA. Possible mechanisms of mature miRNA downregulation involve inhibition of mature miRNA biogenesis and induction of mature miRNA degradation. In accordance, a recent *in vitro* study showed that DNA/LNA mixmer-type antimiRs bound primary transcript of miRNA (53). However, the present *in vivo* experiments with HDO-antimiR did not show suppression of target primary-miRNA biogenesis (Figure 5G), indicating that the reductions of total miR-122 by HDO-antimiR reflect induced degradation of mature miRNA.

As we explain before, antimiRs that include nucleotides with higher binding affinity, such as LNA, likely dose not degrade bound miRNAs (20,21). However, it is interesting that the antimiR gains the ability to degrade the target miRNA when hybridized with the complementary RNA strand, although unwound antimiR strand from HDO-antimiR is the same antimiR as the parent single-stranded antimiR. This degradation may be associated with a different intracellular pathway induced by a change of binding patterns with intracellular proteins due to the double-stranded structure. The intracellular mechanism of HDO-antimiR is indicated by two findings in this study as follows. First, FRET-imaging analysis shows the endosomal escape of double-stranded HDO-antimiR without unwinding (Supplementary Figure S10), indicating that HDO-antimiR partially keeping double-strands would interact with intracellular proteins. Secondly, EMSA analysis shows the change of binding patterns with serum proteins (Figure 3C), suggesting a change of binding patterns with intracellular proteins as well. In addition, this degradation mechanism may lead an increased catalysis of target miRNA. However, more works are required to conclude that this degradation-mechanism is responsible for the increased potency with HDO-antimiR,

In this study, HDO-antimiR silenced miR-21 more efficiently than the parent antimiR in various tissues (Supplementary Figure S2). This finding indicates an advantage that miRNA silencing by HDO-antimiR can be applied to multiple tissue types and also a disadvantage that target tissue specificity may be limited. In previous studies, hepatocyte specificity and efficiency of siRNA (60) and ASO (39,40) are improved by conjugation with a delivery ligand, GalNAc. Our experiments using HDO-antimiR conjugated with the lipid ligand, tocopherol and GalNAc, showed improved miRNA-silencing efficiency of HDO-antimiR in liver tissues (Supplementary Figure S1 and S4). This enhancement of potency likely reflects not only increasing invasion of RISC associated with the target miR (54,61,62) but also improved hepatic delivery, as shown in previous studies of liver targeting following ligand-conjugation to ASO (31,35). Hence, delivery ligands can lead to higher efficiency and also tissue specificity of HDO-antimiR technology.

We have several limitations in this study. Although the detection of the targeted miR-122 by qRT-PCR analysis is inhibited by antimiR administration in dose-dependent and sequence specific manners, qRT-PCR analysis may detect not all functional miRNA (36). We found that both HDO-antimiR with and without Cy3 have similar profiles of binding to serum molecules with EMSA analysis (data not shown). However, there remains a possibility that Cy3 modulate the protein binding properties according to a previous paper (63).

In conclusion, herein we developed a novel antimiR molecular structure, and demonstrate the greater intracellular miRNA-silencing potency of the HDO-antimiR, indicating that the present HDO-technology is a new platform in the field of miRNA regulation.

## Supplementary Material

gkz492_Supplemental_File

## Notes

Present address: Takanori Yokota, Department of Neurology and Neurological Science, Tokyo Medical and Dental University (TMDU), 1-5-45 Yushima, Bunkyo-Ku, Tokyo 113-0034, Japan.
