# Supplementary material for: Highly efficient silencing of microRNA by heteroduplex oligonucleotides
Source: Nucleic Acids Res. 2019 Jun 19;47(14):7321–32. doi: 10.1093/nar/gkz492 (PMC6698647; doi:10.1093/nar/gkz492)
Supplement: gkz492_Supplemental_File [file gkz492_supplemental_file.docx]

Supplementary material for

**Highly efficient silencing of microRNA by heteroduplex oligonucleotides**

Kotaro Yoshioka^1, *^, Taiki Kunieda^1, *^, Yutaro Asami^1, *^, Huijia Guo^1, *^, Haruka Miyata^1^, Kie Yoshida-Tanaka^1^, Yumiko Sujino^1^, Wenying Piao^1^, Hiroya Kuwahara^1^, Kazutaka Nishina^1^, Rintaro Iwata Hara^1, 2^, Tetsuya Nagata^1^, Satoshi Obika^3^, Takeshi Wada^2^ and Takanori Yokota^1,#^

^1^ Department of Neurology and Neurological Science, Tokyo Medical and Dental University, 1-5-45, Yushima, Bunkyo-Ku, Tokyo, 113-8519, Japan

^2^ Faculty of Pharmaceutical Sciences, Tokyo University of Science, 2641 Yamazaki, Noda, Chiba, 278-8510, Japan

^3^ Graduate School of Pharmaceutical Sciences, Osaka University, 1-6 Yamadaoka, Suita-shi, Osaka, 565-0871, Japan

**Supplementary Figure 1**

**

*

**

*

*

**Silencing effects of the parent or HDO-antimiRs with or without conjugation of tocopherol ligand in liver tissues**

qRT-PCR analysis of miR-122 level in livers from mice treated with the single-stranded without or with tocopherol (Toc) conjugation or HDO-antimiR without or with Toc-conjugation to its cRNA at three days after single intravenous injections at 24 nmol/kg; data are normalized to *sno234* RNA expression and presented as means ± SEM.; n = 4; **P* < 0.05, ***P* < 0.01; multiple comparisons were performed using one-way ANOVA with Bonferroni’s test.

**Supplementary Figure 2**

**A**

**B**

*

**

*

**Silencing effects of HDO-antimiR targeting miR-21 on target mRNA in liver tissues**

(A) qRT-PCR analysis of relative *spastic paraplegia 20* (*Spg20*) and (B) *TATA-box binding protein associated factor 7* (*Taf7b*) mRNA expression which are suppressed by miR-21 in the livers of mice treated with antimiR or HDO-antimiR against miR-21 (or the PBS control) at three days after single intravenous injections of 5 or 20 nmol/kg; data are normalized to *Actb* (beta-actin) mRNA and presented as means ± SEM.; n = 5; **P* < 0.05, ***P* < 0.01; multiple comparisons were performed using one-way ANOVA with Bonferroni’s test.

**Supplementary Figure 3**

**

**A**

*

**B**

*

**C**

*

**Silencing effect on mRNA in extrahepatic tissues**

qRT-PCR analysis of *Taf7* mRNA expression in kidney (A), adrenal gland (B) and spleen (C) tissues from mice treated with the parent antimiR or HDO-antimiR against miR-21 (or the PBS control) at three days after single 1980- or 7930-nmol/kg intravenous injections; data are normalized to *Actb* mRNA and presented as means ± SEM.; n = 5, **P* < 0.05, ***P* < 0.01. Multiple comparisons were performed using one-way ANOVA with Bonferroni’s test.

**Supplementary Figure 4**

n.s.

***

**Silencing effects of the parent or HDO-antimiRs with conjugation of GalNAc ligand in liver tissues**

qRT-PCR analysis of miR-122 level and antimiR concentration in livers from mice treated with the single-stranded antimiR with *N*-acetylgalactosamine (GalNAc) conjugation (GalNAc-antimiR) or HDO-antimiR with GalNAc to its cRNA (GalNAc-HDO) at three days after single intravenous injections at 1.5 nmol/kg; data are normalized to *U6* snRNA expression and presented as means ± SEM.; n = 4; ***P < 0.001; multiple comparisons were performed using one-way ANOVA with Bonferroni’s test.

**Supplementary Figure 5**

Serum (-)

*

*

Serum (+)

**

**

**Silencing effects of the parent or HDO-antimiRs by gymnotic delivery with or without serum.**

qRT-PCR analysis of *Spg20* or *Taf7* mRNA expression which are suppressed by miR-21 in Hepa-1-6 cells at 48 h after treatment with increasing concentrations of naked antimiR or HDO-antimiR targeting miR-21 without (upper panels) or with 30 % mouse serum (lower panels).; data are normalized to GAPDH mRNA expression and presented as means ± SEM.; n = 3; *P < 0.05, **P < 0.01.

**Supplementary Figure 6**

**
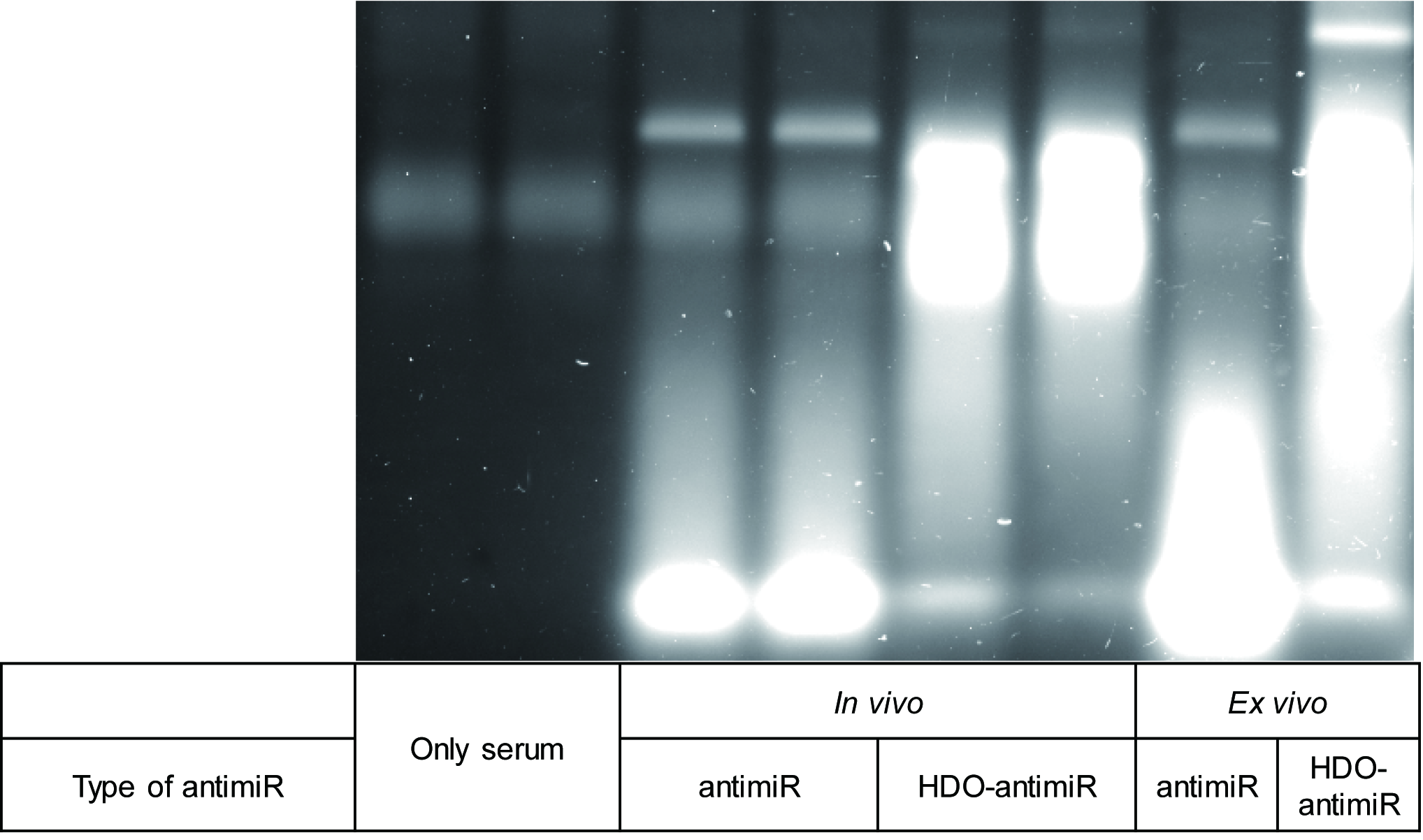
**

**EMSA analysis with *in vivo* or *ex vivo* serum samples**

EMSA analyses showing binding of Cy3–labeled antimiR or HDO-antimiR (2’OMe6 PS6) to serum molecules were performed using serum from mice at ten min after intravenous injection (*in vivo*) or incubation with mouse serum (*ex vivo*).

**Supplementary Figure 7**

**
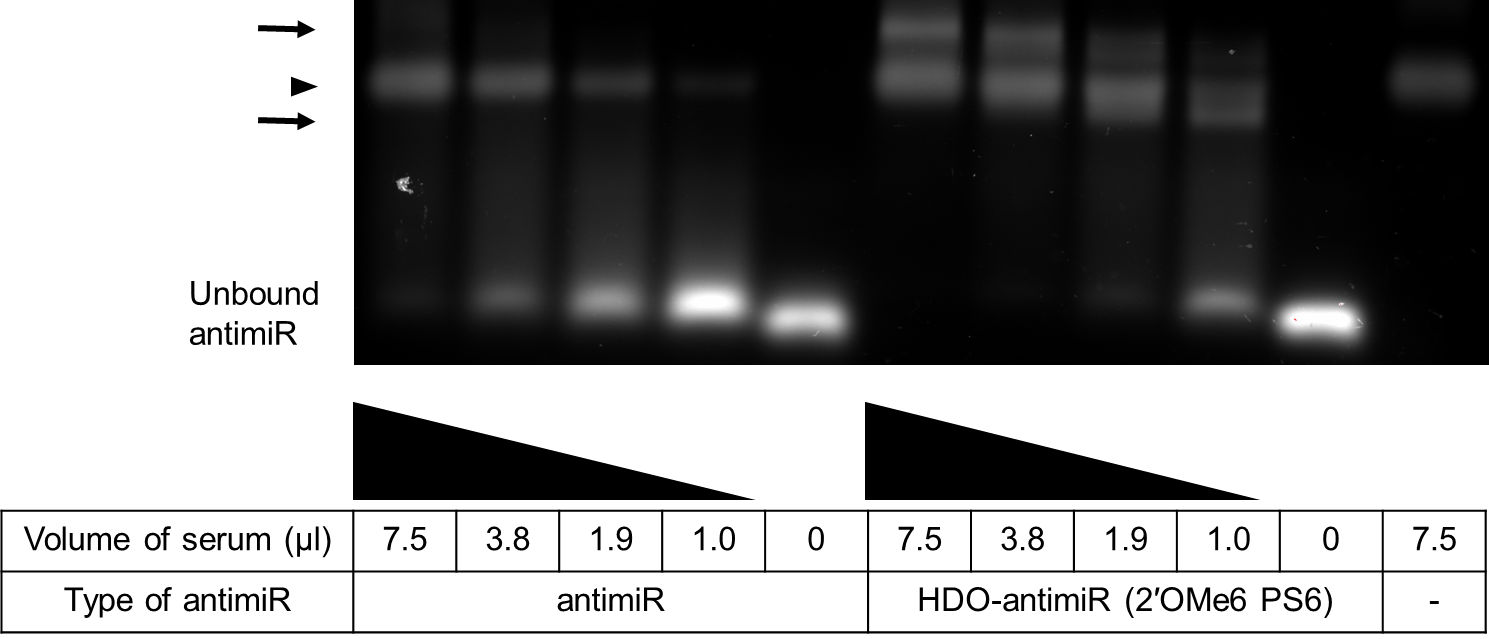
Electrophoretic mobility shift assay (EMSA) analysis after incubation with human serum**

EMSA analysis showing binding of Cy3–labeled antimiR or HDO-antimiR (2’OMe6 PS6) to serum molecules after incubation with increasing concentrations of human serum in PBS. The positions of bands corresponding with complexes of parent antimiR and HDO-antimiR are indicated by an arrow head and arrows, respectively.

**Supplementary Figure 8**


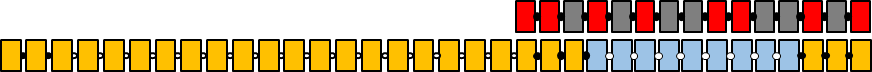

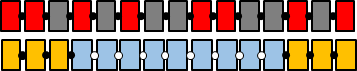


**Silencing effect of HDO-antimiR with an overhanging portion of the cRNA**-**strand**

qRT-PCR analyses of miR-122 in liver tissues from mice treated with HDO-antimiR (2’OMe6 PS6; 15/15mer-HDO), HDO-antimiR with a cRNA-strand overhanging to 35mer (15/35mer-HDO) or PBS (control) at three days after single 24-nmol/kg injections; data are normalized to U6 snRNA and are presented as means ± SEM; n = 5.

**Supplementary Figure 9**

**
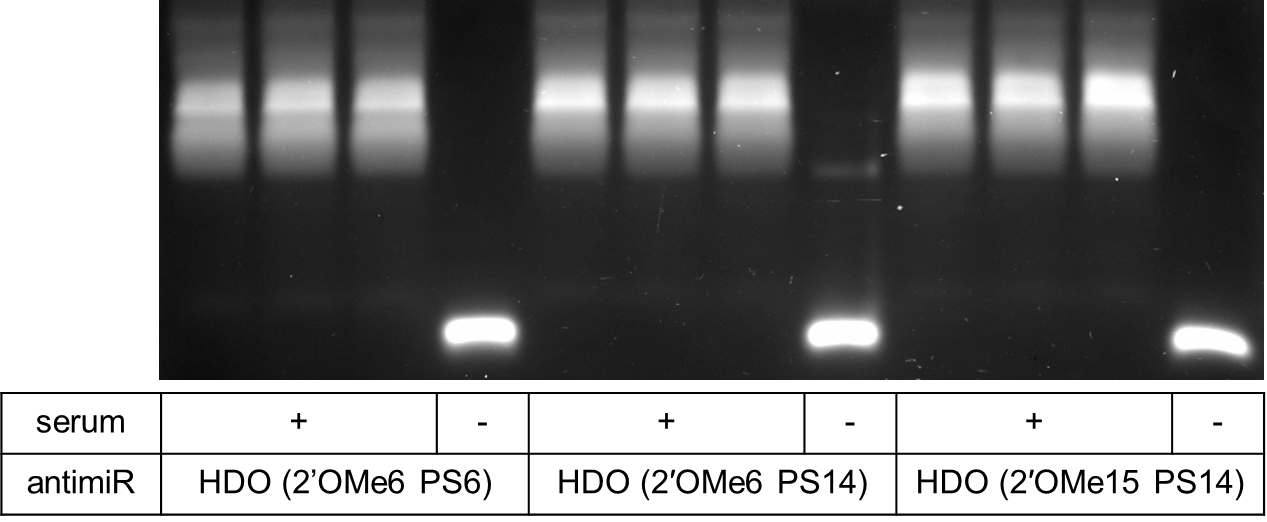
EMSA analysis of HDO-antimiRs with full PS or OM modifications of the cRNA**-**strand**

EMSA analysis showing binding of Cy3–labeled HDO-antimiRs (2’OMe6 PS6, 2’OMe6 PS14, 2’OMe15 PS14) to serum molecules after incubation with mouse serum.

**Supplementary Figure 10**

**A**


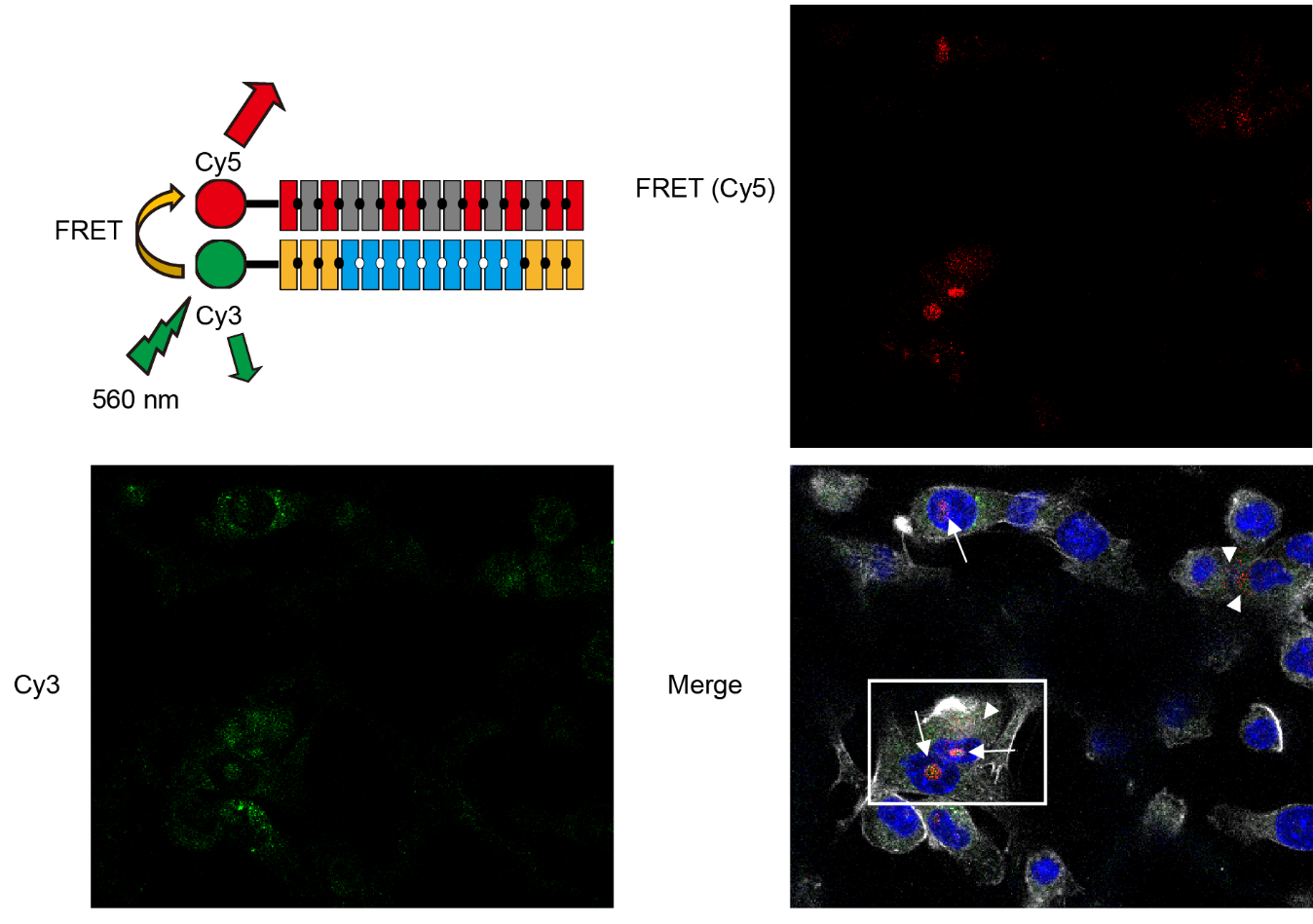


**B**

**
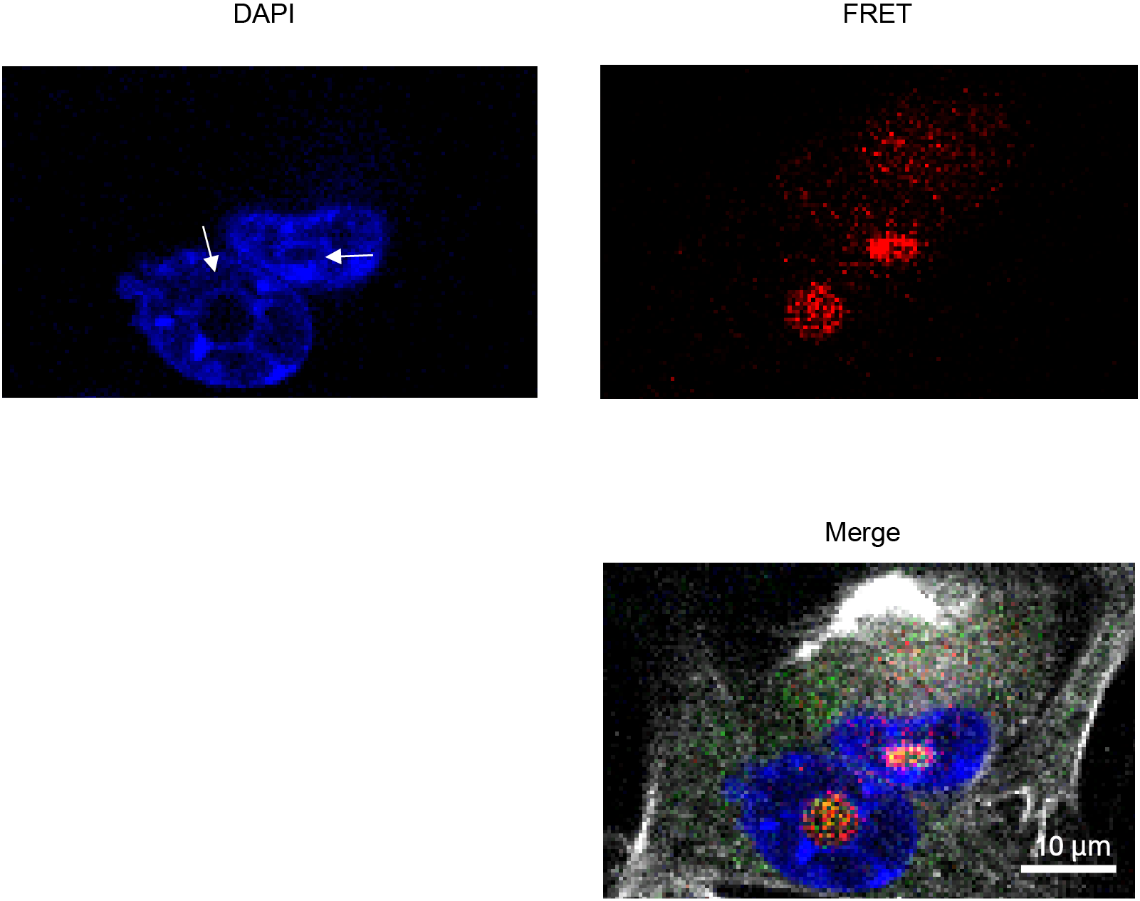
**

**C**

**
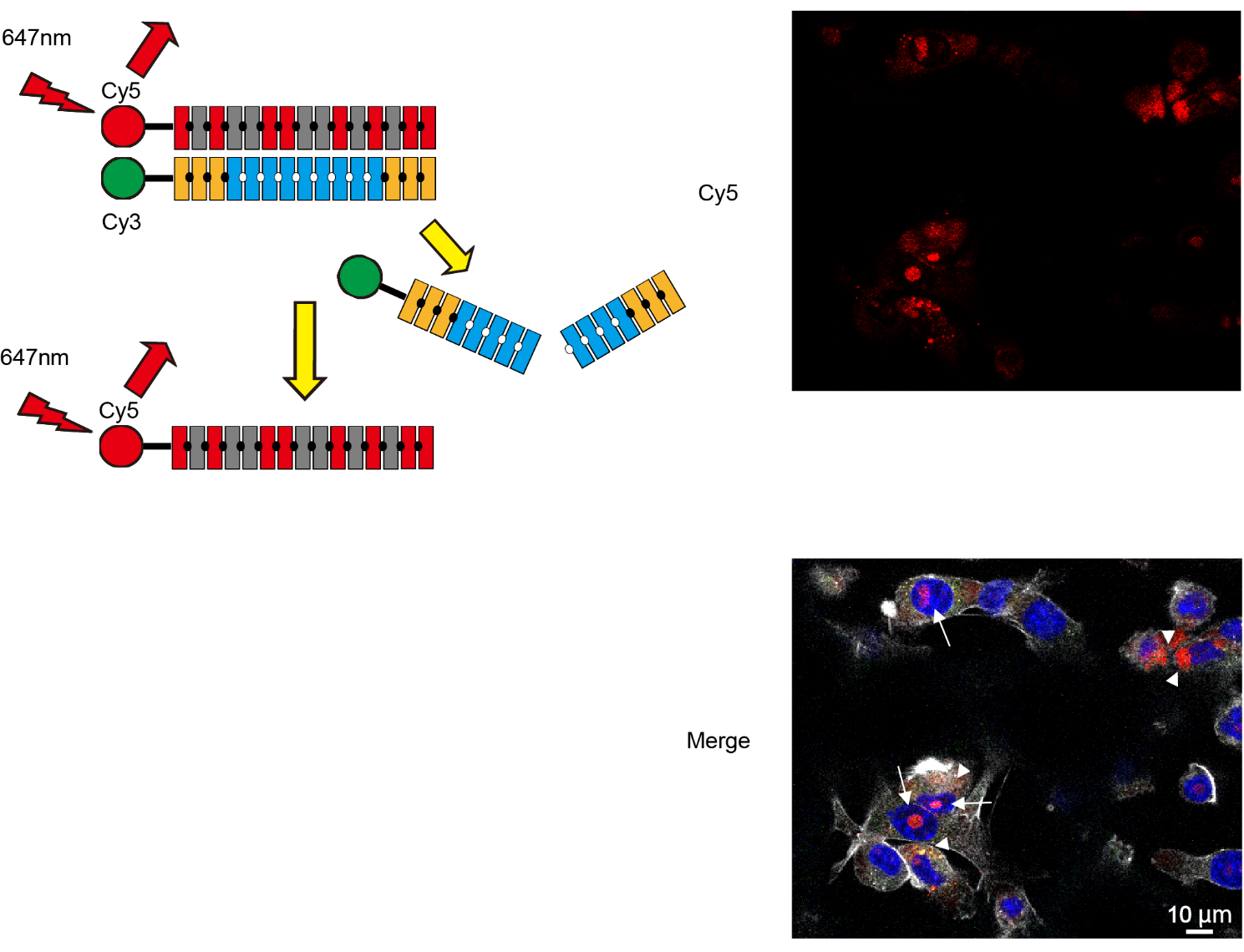
**

**FRET-based imaging analyses of intracellular unwinding and trafficking of HDO-antimiR**.

(A-B) Cy3 (donor) in the cRNA strand and Cy5 (acceptor) in the antimiR strand are a FRET pair, and the proximity of these dyes on the double-stranded HDO-antimiR results in FRET. FRET (Cy5): excitation 560 nm, emission 663–738 nm [red]; Cy3: excitation 560 nm, emission 570-620 nm [green]; AlexaFluor 488 phalloidin [white]; DAPI [blue]; FRET signal in nucleus (arrow) and in cytoplasm (arrow head). White box in (A) stands for zoomed area in (B). Arrows in (B) indicate nucleolus. (C) Confocal imaging for Cy5 in the antimiR-strand of HDO-antimiR. Cy5: excitation 647 nm, emission 663–738 nm.

**Supplementary Figure 11**


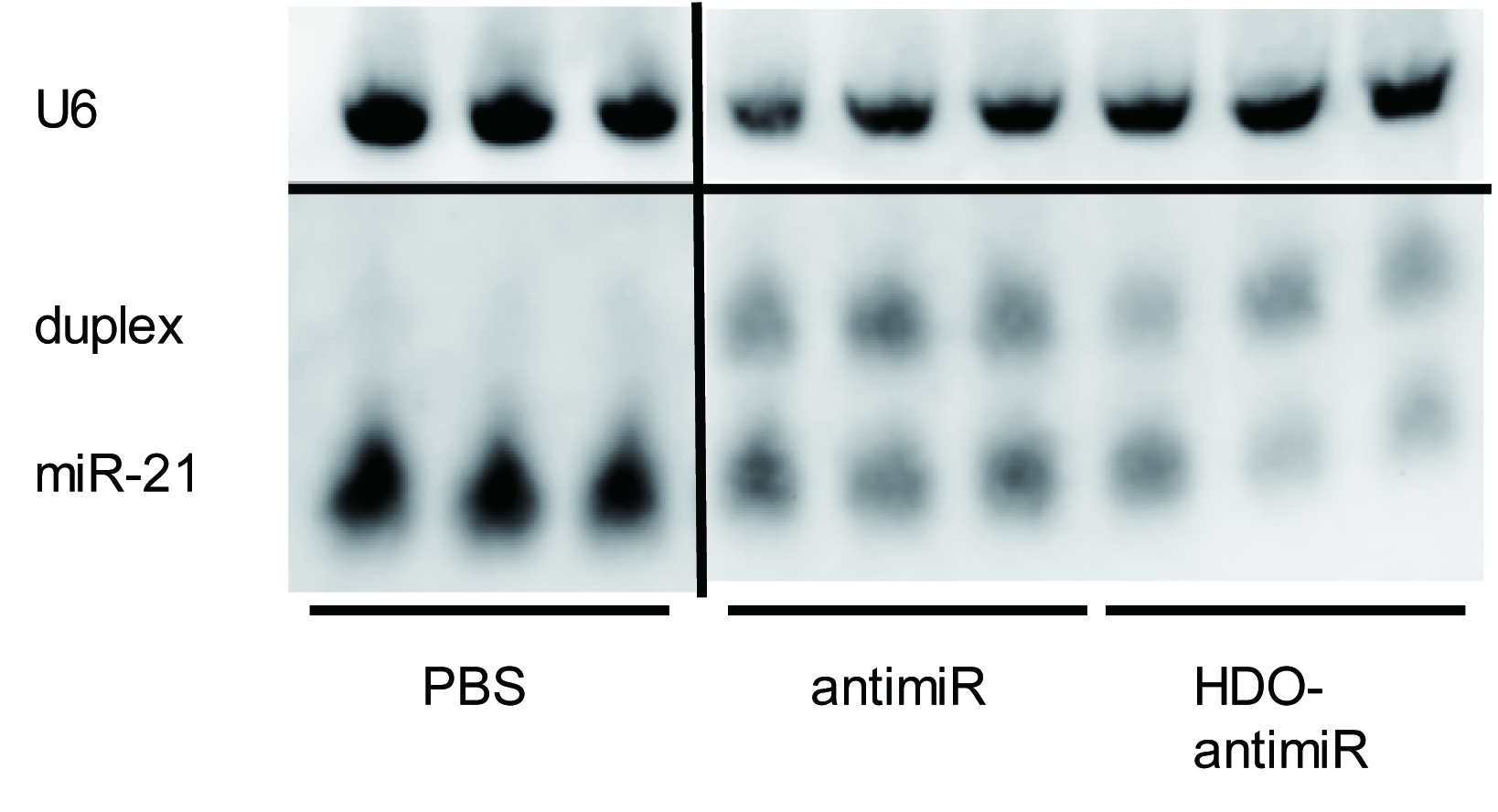


**Efficacy and pattern of miRNA inhibition by HDO-antimiR targeting miR-21 on northern blot**

Northern blotting analyses with a miR-21-specific probe using RNA samples from livers of mice at 3 days after treatment with injections of 20 nmol/kg parent antimiR, HDO-antimiR targeting miR-21, or PBS (n = 3).

**Supplementary Table 1. Oligonucleotide sequences**

| Name | Sequence |
| --- | --- |
| miR-122 targeting |  |
| DNA/LNA antimiR-122 | c*c*a*t*t*g*t*c*a*c*a*c*t*c*c |
| cRNA (2′OMe6 PS6) | G*G*A*GUGUGACAA*U*G*G |
| cRNA (2′OMe0 PS0) | GGAGUGUGACAAUGG |
| cRNA (2′OMe6 PS0) | GGAGUGUGACAAUGG |
| cRNA (2′OMe0 PS6) | G*G*A*GUGUGACAA*U*G*G |
| cRNA overhanging (2′OMe26 PS8) | G*G*A*GUGUGACAA*U*G*GAUGCGUAGCAUUGGUAUU*C*A |
| cRNA overhanging (2′OMe35 PS16) | G*G*A*G*U*G*U*G*A*C*A*A*U*G*GAUGCGUAGCAUUGGUAUU*C*A |
| cRNA (2′OMe6 PS14) | G*G*A*G*U*G*U*G*A*C*A*A*U*G*G |
| cRNA (2′OMe15 PS14) | G*G*A*G*U*G*U*G*A*C*A*A*U*G*G |
| miR-21 targeting |  |
| DNA/LNA antimiR-21 | t*c*a*g*t*c*t*g*a*t*a*a*g*c*t |
| cRNA (2′OMe6 PS6) | A*G*C*UUAUCAGAC*U*G*A |

Lower case letters represent DNA; lower case underlined represents LNA; c denotes LNA 5-methylcytosine; upper case represents RNA, upper case underlined represents 2′-O-methyl sugar modification; * indicates phosphorothioate internucleotide linkage.

**Supplementary Table 2. Taqman primers information**

| Gene name | Catalog number |
| --- | --- |
| miR-122 | 002245 |
| pri miR-122 | Mm03306556_pri mmu-mir-122 |
| miR-21 | 000397 |
| U6 snRNA | 001973 |
| Aldoa | Mm00833172_g1 |
| Bckdk | Mm00437777_m1 |
| Taf7 | Mm00558925_s1 |
| Spg20 | Mm01289634_m1 |
| Actb | Mm00607939_s1 |

**Supplementary Table 3. Serum, liver, and kidney parameters of mice after treatment with PBS (control), antimiR, or HDO-antimiR**

*

|  | LDL  (mg/dL) | AST (U/L) | ALT (U/L) | Total bilirubin (mg/dL) | Blood urea nitrogen (mg/dL) | Creatinine (mg/dL) |
| --- | --- | --- | --- | --- | --- | --- |
| PBS | 11.4 ± 0.6 | 76 ± 5 | 31 ± 3 | 0.064 ± 0.002 | 30.7 ± 1.5 | 0.124 ± 0.011 |
| antimiR |  |  |  |  |  |  |
| 0.70 µmol/kg | 9.8 ± 1.0  *** | 65 ± 4 | 29 ± 3 | 0.054 ± 0.005 | 26.6 ± 1.3 | 0.144 ± 0.010 |
| HDO-antimiR | **** |  |  |  |  |  |
| 0.70 µmol/kg | 4.8 ± 1.0 | 56 ± 4 | 28 ± 3 | 0.048 ± 0.005 | 24.0 ± 1.7 | 0.130 ± 0.010 |

Serum samples were collected at seven days after three injections in a week. Data are presented as means ± SEM; *n* = 5; **P* < 0.05, ****P* < 0.001, *****P* < 0.0001 (Bonferroni’s multiple-comparison test)
